# Oxidative metabolism of astrocytes is not reduced in hepatic encephalopathy: a PET study with [^11^C]acetate in humans

**DOI:** 10.3389/fnins.2014.00353

**Published:** 2014-11-03

**Authors:** Peter Iversen, Kim Mouridsen, Mikkel B. Hansen, Svend B. Jensen, Michael Sørensen, Lasse K. Bak, Helle S. Waagepetersen, Arne Schousboe, Peter Ott, Hendrik Vilstrup, Susanne Keiding, Albert Gjedde

**Affiliations:** ^1^Department of Nuclear Medicine and PET Centre, Aarhus University HospitalAarhus, Denmark; ^2^Center of Functionally Integrative Neuroscience, Aarhus UniversityAarhus, Denmark; ^3^Department of Nuclear Medicine, Aalborg University HospitalAalborg, Denmark; ^4^Department of Chemistry and Biochemistry, Aalborg UniversityAalborg, Denmark; ^5^Department of Hepatology and Gastroenterology, Aarhus University HospitalAarhus, Denmark; ^6^Department of Drug Design and Pharmacology, Faculty of Health and Medical Sciences, University of CopenhagenCopenhagen, Denmark; ^7^Brain Research and Integrative Neuroscience Laboratory, Department of Neuroscience and Pharmacology, Faculty of Health and Medical Sciences, University of CopenhagenCopenhagen, Denmark

**Keywords:** astrocytes, brain energy metabolism, kinetic modeling, mitochondria, positron emission tomography

## Abstract

In patients with impaired liver function and hepatic encephalopathy (HE), consistent elevations of blood ammonia concentration suggest a crucial role in the pathogenesis of HE. Ammonia and acetate are metabolized in brain both primarily in astrocytes. Here, we used dynamic [^11^C]acetate PET of the brain to measure the contribution of astrocytes to the previously observed reduction of brain oxidative metabolism in patients with liver cirrhosis and HE, compared to patients with cirrhosis without HE, and to healthy subjects. We used a new kinetic model to estimate uptake from blood to astrocytes and astrocyte metabolism of [^11^C]acetate. No significant differences of the rate constant of oxidation of [^11^C]acetate (*k*_3_) were found among the three groups of subjects. The net metabolic clearance of [^11^C]acetate from blood was lower in the group of patients with cirrhosis and HE than in the group of healthy subjects (*P* < 0.05), which we interpret to be an effect of reduced cerebral blood flow rather than a reflection of low [^11^C]acetate metabolism. We conclude that the characteristic decline of whole-brain oxidative metabolism in patients with cirrhosis with HE is not due to malfunction of oxidative metabolism in astrocytes. Thus, the observed decline of brain oxidative metabolism implicates changes of neurons and their energy turnover in patients with HE.

## Introduction

Hepatic encephalopathy (HE) is a common and recurrent complication of liver cirrhosis. Increased blood ammonia is held to be a key pathogenic factor (Ott and Vilstrup, 2014). Compared to patients with cirrhosis without HE and healthy subjects, patients with cirrhosis and HE type C had low cerebral oxygen metabolism (CMRO_2_) and cerebral blood flow (CBF) in inverse proportion to arterial blood ammonia concentration (Iversen et al., 2009; Dam et al., 2013). Both variables rise to normal after recovery from HE (Dam et al., 2013), indicating that low values of CMRO_2_ and CBF during HE are related to the encephalopathy rather than to the cirrhosis. Oxygen delivery to the brain by the circulation did not limit the magnitude of CMRO_2_, suggesting that the reduction of CBF is secondary to the reduction of CMRO_2_ (Iversen et al., 2009; Gjedde et al., 2010). In brain, ammonia reacts with glutamate dehydrogenase and glutamine synthetase to form glutamate and glutamine, respectively, the former of which is linked to oxidative metabolism. The disposal of ammonia in the brain takes place mainly via formation of glutamine by glutamine synthetase which is located exclusively in astrocytes. This is the basis for the present hypothesis that the reduction in CMRO_2_ during cirrhosis with HE reflects reduced oxidative metabolism in astrocytes.

Acetate is converted to acetyl-CoA and undergoes subsequent oxidative metabolism in the TCA cycle of most cells. However, in the brain, acetate is metabolized in the astrocytes because the monocarboxylate transporter 2 (MCT2), the only MCT of neurons, fails to recognize acetate to the same extent as MCT1 located in endothelial cells and astrocytes (Waniewski and Martin, 1998; Patel et al., 2010). Therefore, measurement of acetate metabolism commonly is used to assess the oxidative metabolism of astrocytes (Patel et al., 2010). In the present study, we used positron emission tomography (PET) with tracer [1-^11^C]acetate ([^11^C]acetate) to assess the oxidative metabolism of astrocytes in patients with cirrhosis, with or without HE, as well as in healthy subjects.

Each subject had PET with [^11^C]acetate immediately after the measurements of CMRO_2_ and CBF, published separately (Iversen et al., 2009). A model of cerebral acetate kinetics was developed for the present report. Based on the findings of reduced CMRO_2_ in patients with cirrhosis and HE (Iversen et al., 2009) and the restriction of the ammonia and acetate metabolism to astrocytes, we tested the hypothesis that [^11^C]acetate metabolism is reduced in the patients with HE, as a measure of oxidative metabolism by astrocytes. Failure to uphold the hypothesis would mean that effects of ammonia in astrocytes do not explain the reduced oxidative metabolism measured in HE.

## Materials and methods

### Subjects

According to the design of the study, each of 21 subjects, seven in each of the groups of patients with cirrhosis and HE type C, patients with cirrhosis without HE, and healthy subjects, was scheduled to have an [^11^C]acetate PET study after the completion of measurements of CMRO_2_ and CBF (Iversen et al., 2009; Gjedde et al., 2010). However, only six patients with HE completed the [^11^C]acetate study. In three other subjects admitted to the study, the design could not be fulfilled because of technical failure of one of the three successive PET measurements. The [^11^C]acetate measurements could be completed in only five of the seven healthy subjects. In the group of patients with cirrhosis, one of the seven patients was unable to inhale [^15^O]oxygen and was excluded from the previous presentation of the oxygen and blood flow data. Thus, six patients with HE, seven patients with cirrhosis without HE, and five healthy subjects completed the [^11^C]acetate studies. Clinical and laboratory characteristics therefore differ in minor respects from those given in the oxygen consumption and blood flow paper (Iversen et al., 2009; Gjedde et al., 2010) (Table 1). At the time of the study, the patients received no specific treatment for HE.

**Table 1:** **Patient Characteristics (revised from Iversen et al., 2009)**.

	**Cirrhosis with HE (*n* = 6)**	**Cirrhosis without HE (*n* = 7)**	**Healthy subjects (*n* = 5)**
Female/Male	1/5	1/6	1/4
Age (years)	58 (48–62)	54 (45–63)	54 (46–65)
Body weight (kg)	75 (45–103)	86 (62–107)	83 (69–89)
MAP (mmHg)	87^*^(81–92)	90^*^(73–107)	106 (102–112)
**HEPATIC ENCEPHALOPATHY**			
Arterial ammonia (μmol/l)	129 ± 64^*^	76 ± 25^*^	21 ± 6
New haven coma grade	I:3; II:1; III:2	None	None
Glasgow coma score	10:1; 12:2; 14:1; 15:2	15:7	15:5
Continuous reaction time (Index)	0.79 (0.33–1.93)^*^^,^^**^	2.05 (0.98–3.02)	2.38 (2.14–2.76)
**ARTERIAL BLOOD GASES**			
pH	7.49 ± 0.02^*^^,^^**^	7.46 ± 0.03	7.43 ± 0.03
pO_2_ (kPa)	11.1 ± 1.3	10.0 ± 0.9	11.2 ± 0.9
O_2_ Saturation (%)	97.1± 1.0	95.8 ± 1.4	97.3 ± 1.5
pCO_2_ (kPa)	4.3 ± 0.7^*^	4.9 ± 0.4	5.4 ± 0.4
SBC (mmol/l)	26.2 ± 2.7	27.0 ± 2.1	26.4 ± 1.6
**METABOLIC MARKERS**			
Plasma glucose (mmol/l)	6.3 ± 1.9	6.2 ± 1.7	5.3 ± 0.4
Plasma ketone bodies (mmol/l)	0.2 ± 0.1	0.3 ± 0.1	0.3 ± 0.3
**LIVER TESTS**			
Child-Pugh class	A:0; B:1; C:5	A:2; B:4; C:1	None
GEC: Pt/Ref^†^	0.51 (0.40–0.61)	0.56 (0.45–0.68)	1.00 (0.85–1.15)

*HE, hepatic encephalopathy; n, number of subjects; MAP, mean arterial pressure*.

† *GEC, galactose elimination capacity is shown as patient GEC relative to GEC for a healthy subject of same age and body weight*.

*Values are given as median (range) or mean ± SD*.

* *Mean value is significantly different from mean of healthy subjects (P < 0.05)*.

** *Mean value is significantly different from mean of cirrhosis without HE (P < 0.05)*.

### Ethics

The study was performed in accordance with the Helsinki II Declaration and approved by the Ethics Committee of Aarhus County. We obtained informed consent from each participant. The radiation dose averaged 2.45 mSv ([^11^C]acetate).

### Tracer production

[^11^C]Carbon dioxide was produced in the Aarhus PET Center's 16.8 MeV PETtrace cyclotron (GE Medical Systems, Uppsala, Sweden) and a slight modified procedure of Roeda et al. (2002) was applied to convert [^11^C]carbon dioxide to [^11^C]acetate. Briefly, we trapped ^11^CO_2_ in a 10-ml vial containing methylmagnesium chloride (1 ml, approximately 100 mmol/l). After complete trapping, we added sterile acetic acid (9 ml, 1 mmol/l) to quench the Grignard solution. We purified the product by passing it over H^+^ and Ag^+^ cartridges and then trapped the [^11^C]acetate on an OH cartridge. We washed the product twice with 10 ml of sterile water. Finally, we used 10 ml of sterile citrate buffer (pH 4.7) to elute the [^11^C]acetate from the OH cartridge through a 0.22 μm filter into a sterile product vial. We measured the activity of the final product and used a sterile needle and syringe to withdraw approximately 0.3 ml of the formulation for quality control. Typical synthesis time was 7 min (±2 min) with a yield of 6 GBq (±2 GBq) after EOS with 10 min bombardment with 40 μA.

### PET recordings

The subjects were told not to take drugs or food for 8 h before the studies but were free to drink water. Catheters were placed percutaneously in a radial artery for blood sampling and in a cubital vein for intravenous injections of the tracers. The subjects were placed in the supine position with their head within the 15 cm field-of-view of the tomograph (ECAT EXACT HR 47, CTI, Knoxville, TN), equipped with a neck shield as previously described (Iversen et al., 2009).

In each subject, the PET recording with [^11^C]acetate took place after completion of the measurements of CMRO_2_ and CBF (Iversen et al., 2009). A median dose of 459 MBq (range 128–580 MBq) [^11^C]acetate were given as intravenous injection during the initial 20 s of a dynamic PET recording of the brain using a protocol that comprised 50 frames of 18 × 5 s, 12 × 10 s, 7 × 30 s, 1 × 120 s, and 12 × 180 s for a total of 45 min. Attenuation correction, radioactivity decay correction and reconstruction procedures were as described previously (Iversen et al., 2009).

### Blood samples

During the [^11^C]acetate PET recordings, we manually collected 0.5 ml arterial blood samples at the following intervals: 18 every 5th s, 9 every 10th s, 12 every 60th s, and every 5th min for a total of 45 min. Blood [^11^C] radioactivity concentrations were measured in a well counter (Packard Instruments Co., Meriden, CT, USA), cross-calibrated with the tomograph and corrected for radioactive decay to the start of the scanning. Arterial plasma concentration of ammonia (van Anken and Schiphorst, 1974) was significantly higher in the patients than in healthy subjects and tended to be higher in the patients with HE than in the patients without HE, although this difference was not significant (Table 1).

### Magnetic resonance imaging

T1-weighted magnetic resonance imaging (MRI) was performed in each subject for definition of specific brain regions for the PET measurements by co-registration as previously described (Iversen et al., 2009).

### Tracer kinetic analysis

**Figure 1:**
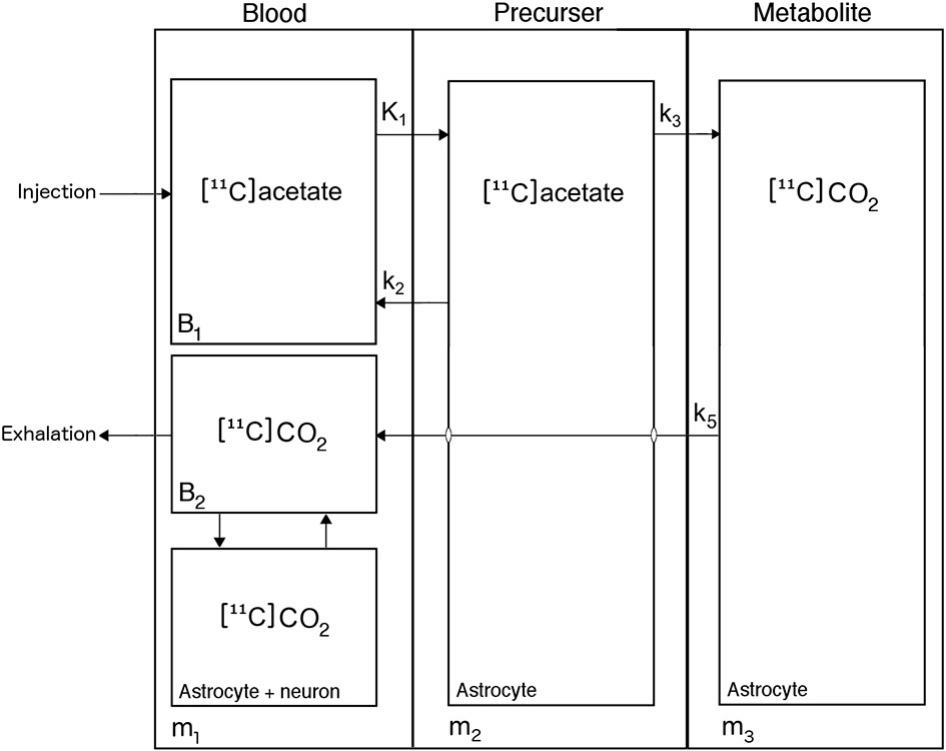
**Kinetic model of [^11^C]acetate metabolism in human brain**. [^11^C]Acetate enters the astrocytes from blood through either carrier-mediated transport (MCT1) or passive diffusion, characterized by the unidirectional clearance *K*_1_, [^11^C]Acetate in the astrocytes either return to blood, characterized by the rate constant *k*_2_, or is converted to [^11^C]acetyl-CoA, thereby entering the tricarboxylic acid (TCA) cycle, where it is joined into amino acids produced from oxaloacetate and α-ketoglutarate, irreversible processes characterized by the rate constant *k*_3_, before it is converted to [^11^C]CO_2_, which leaves the cell by passive diffusion into the blood, characterized by the rate constant *k*_5_.

The kinetic analysis of [^11^C]acetate uptake from blood and metabolism in astrocytes is complicated by rapid metabolism of [^11^C]acetate to [^11^C]CO_2_. This requires a model that includes three successive steps: transport of [^11^C]acetate from blood to brain cells (*K*_1_; ml blood (cm^−3^ brain tissue) min^−1^), backflux from cells to blood (rate constant *k*_2_; min^−1^), metabolism of [^11^C]acetate to [^11^C]acetyl-CoA and [^11^C]CO_2_ (*k*_3_; min^−1^), flux of [^11^C]CO_2_ out of the brain to the blood circulation (*k*_5_; min^−1^) and specifically into the apparent distribution volume in the vascular space (*V*_0_; ml blood (cm^−3^ brain tissue)) (Figure 1). We symbolize the volume of distribution of labeled molecules remaining in the vascular bed of the brain by the term *V*_0_, assumed to include effects of labeled tracer metabolites such as [^11^C]CO_2_, as described below. As the [^11^C]acetate conversion to [^11^C]CO_2_ is irreversible, the model has no rate constant for the conversion of ^11^C-labeled metabolites to the [^11^C]acetate itself. The constant *k*_3_ is a measure of the rate constant for the oxidative metabolism of blood-borne [^11^C]acetate in the astrocytes. We note that this may be different from the net whole-brain metabolic clearance of acetate.

We used a statistical non-linear mixed effects model to estimate the parameters of the kinetic model *K*_1_*k*_2_*k*_3_*k*_5_*V*_0_ (Williams and Ette, 2000) described by the following equations:

$$\begin{array}{l} m_{1}=V_{o}\;c_{1}\\ m_{2}=K_{1}\int_{0}^{T}c_{1}\;e^{-\left(k_{2}+k_{3}\right)\left(T-t\right)}\;dt\\ m_{3}=K_{1}k_{3}\int_{0}^{u}\left[\int_{0}^{T}c_{1}\;e^{-\left(k_{2}+k_{3}\right)\left(T-t\right)}\;dt\right]e^{-k_{5}\left(u-T\right)}\;dt \end{array}$$

where *m*_1_, *m*_2_, and *m*_3_ are the tracer quantities in compartments 1–3 (Figure 1); *c*_1_(*t*) is the arterial tracer concentration of compartment 1 as function of time t after onset of recording; and *u* and *T* are dummy time variables. This approach avoids “overfitting” to the measured data in individual subjects by simultaneously fitting the model to all subjects, admitting different kinetic parameters in each of the three groups of subjects but allowing only modest deviations within each group. The latter was ensured by modeling subjects as random effects. In contrast, a two-step procedure with individual model fitting followed by parameter averaging and group testing may lead to “overfitting” and variance inflation in the first step and in the second step. The procedure overcomes a potentially poor match between the group-models with averaged kinetic coefficients compared to individual time activity curves that may occur when the averaging step is performed irrespectively and independently of the measured data.

The kinetic model was fitted to the PET data from whole-brain and each of nine predefined brain regions of each subject (cerebellum, frontal, parietal, temporal and occipital lobes, striatum, thalamus, total gray matter, and total white matter), see Results. We obtained estimates of rate constants and clearances defined by the model, including the net metabolic clearance of [^11^C]acetate *K* as *K*_1_*k*_3_/(*k*_2_ + *k*_3_) (Gjedde, 1982) listed in Table 2. We also estimated the *k*_3_/*k*_5_ ratio as an oxidative index, expressing the relative rates of oxidative metabolism in astrocytes and flow-dependent removal of [^11^C]-metabolites from brain tissue to blood.

**Table 2:** **Parameters of brain [^11^C]acetate metabolism**.

		**Cirrhosis with HE (*n* = 6)**	**Cirrhosis without HE (*n* = 7)**	**Healthy subjects (*n* = 5)**
*K*_1_(ml blood (cm^−3^ brain tissue) min^−1^)	WB	0.24 ± 0.043	0.41 ± 0.040^*^	0.43 ± 0.047^*^
	GM	0.23 ± 0.046	0.41 ± 0.043^*^	0.41 ± 0.051^*^
	WM	0.19 ± 0.026	0.27 ± 0.024^**^	0.23 ± 0.028
*k*_2_ (min^−1^)	WB	0.58 ± 0.061	0.93 ± 0.057^*^	0.90 ± 0.068^*^
	GM	0.58 ± 0.067	0.95 ± 0.062^*^	0.90 ± 0.074^*^
	WM	0.47 ± 0.049	0.61 ± 0.046^**^	0.48 ± 0.054
*k*_3_ (min^−1^)	WB	0.098 ± 0.014	0.094 ± 0.013	0.11 ± 0.015
	GM	0.096 ± 0.014	0.097 ± 0.013	0.11 ± 0.016
	WM	0.130 ± 0.046	0.072 ± 0.041	0.12 ± 0.050
*k*_5_ (min^−1^)	WB	0.087 ± 0.012	0.11 ± 0.012	0.13 ± 0.014^*^
	GM	0.075 ± 0.011	0.11 ± 0.010^*^	0.12 ± 0.013^*^
	WM	0.135 ± 0.031	0.18 ± 0.031	0.17 ± 0.035
*V*_0_ (ml blood (cm^−3^ brain tissue))	WB	0.021 ± 0.014	0.039 ± 0.0129	0.042 ± 0.0153
	GM	0.022 ± 0.014	0.037 ± 0.0138	0.042 ± 0.0163
	WM	0.009 ± 0.008	0.019 ± 0.0076	0.013 ± 0.0090
*K* (ml blood (cm^−3^ brain tissue) min^−1^)	WB	0.034 ± 0.008	0.038 ± 0.0310	0.047 ± 0.0069^**^
	GM	0.033 ± 0.008	0.038 ± 0.0330	0.045 ± 0.0060^**^
	WM	0.041 ± 0.013	0.028 ± 0.0240	0.046 ± 0.0180
Oxidative index (*k*_3_/*k*_5_)	WB	1.13 ± 0.23	0.83 ± 0.14	0.84 ± 0.15
	GM	1.27 ± 0.27	0.90 ± 0.15	0.96 ± 0.17
	WM	0.96 ± 0.40	0.41 ± 0.24	0.72 ± 0.33
*K*_1_ ([^15^O]water) (ml blood cm^−3^ brain tissue min^−1^)^†^	WB	0.24 ± 0.030	0.41 ± 0.061^*^	0.43 ± 0.041^*^
	GM	0.26 ± 0.033	0.45 ± 0.072^*^	0.48 ± 0.050^*^
	WM	0.21 ± 0.030	0.31 ± 0.044^*^	0.28 ± 0.011^*^

*HE, hepatic encephalopathy; n, number of subjects; K_1_, unidirectional clearance of [^11^C]acetate transfer from blood to astrocytes; k_2_, rate constant of [^11^C]acetate by return to blood from astrocytes; k_3_, rate constant of [^11^C]acetate by conversion to [^11^C]acetylCoA, thereby entering the tricarboxylic acid cycle (TCA); k_5_, passive diffusion of ^11^CO_2_ into the blood; V_0_, Apparent distribution volume in vascular space; K, net metabolic clearance of [^11^C]acetate; WB, whole brain region excl. ventricles; GM, gray matter region; WM, white matter region*.

*Values are given as mean ± SD*.

* *Mean value is significantly different from mean of the group of patients with cirrhosis and HE (P < 0.01)*.

** *Mean value is significantly different from mean of the group of patients with cirrhosis and HE (P < 0.05)*.

† *Revised from Iversen et al. (2009)*.

We did not make any specific correction for the appearance of [^11^C]CO_2_ in blood. The question of the accumulation of [^11^C]CO_2_ in blood, produced from the metabolism of [^11^C]acetate, has been dealt with on a number of occasions (e.g., Shields et al., 1992). In the accompanying editorial, Gjedde (1992) stated that “[if] the distribution of [^11^C]CO_2_ initially follows blood flow, reflecting rapid transfer into the tissue, it may be sufficient simply to calculate tissue curves that assume instant steady state with the blood, at half the blood level.” After administration of [^11^C]acetate, Shields et al. found that [^11^C]-labeled carbon dioxide, 1–2 min after injection of [^11^C]acetate, represented a constant proportion of about 60% of the [^11^C]-activity in blood, and that brain tissue [^11^C]-activity followed blood [^11^C]-activity at approximately the same level. This means that the carbon dioxide activities in blood and brain tissue kinetically can be attributed to part of the vascular background. In the present analysis, the vascular background volume *V*_0_ was included specifically for this reason. We tested this assertion by confirming the absent relation of the estimates of *V*_0_ to the estimates of the flow-sensitive parameters *K*_1_, *k*_2_, and *k*_5_, and the *V*_0_ estimates' poor relation to the estimates of *k*_3_, which is an indicator of the capacity for metabolism of [^11^C]acetate in brain tissue.

### Statistics

We observed no significant left-right differences for any of the present kinetic parameter estimates in any of the predefined regions, and we therefore report the estimates of the kinetic parameters as averages for right and left brain regions. We used a Two-Way ANOVA test to compare the oxidative indices between groups, with probability values of less than 0.05 as the threshold of statistical significance. We also determined the significance of relations among selected individually estimated rate constants for whole-brain, using linear regression with probability values of less than 0.05 as the threshold of statistical significance.

## Results

The rate constant of oxidation of [^11^C]acetate, *k*_3_, used here to represent the efficiency of oxidative metabolism of [^11^C]acetate in astrocytes, did not reveal any significant differences among the three groups of subjects, in any of the brain regions studied (Table 2).

**Figure 2:**
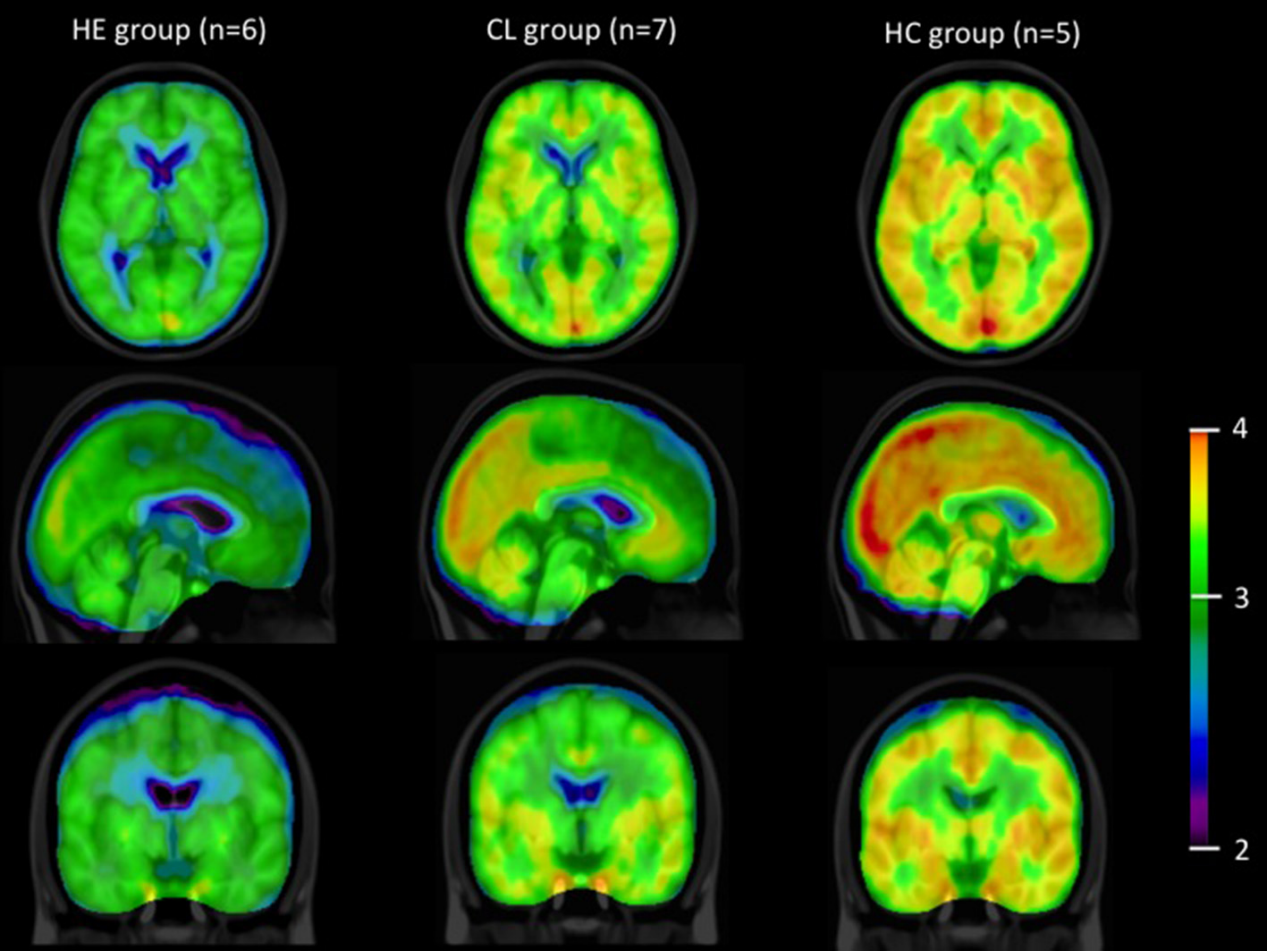
**[^11^C]acetate PET images**. Summed average [^11^C]-PET images from time of injection of [^11^C]acetate to 45 min in the groups of patients with cirrhosis and HE (HE), patients with cirrhosis without HE (CL) and healthy subjects (HC), n, number of subjects. Data are presented on a logarithmic scale.

Figure 2 illustrates the observation that the average summed [^11^C]-activity was lower in the group of patients with cirrhosis and HE compared to the group of patients with cirrhosis without HE and that of healthy subjects. These differences were not significantly related to differences of *k*_3_ among the three groups of subjects, for any brain region.

The group of patients with cirrhosis and HE had lower whole-brain and gray-matter unidirectional clearances *K*_1_ of [^11^C]acetate from blood to brain tissue than the groups of patients without HE and healthy subjects (both *P* < 0.01). There were no significant differences of *K*_1_ between the patients without HE and the healthy subjects. The highest values of *K*_1_ were seen in cerebellum, occipital and parietal cortices, and the lowest values in striatum, thalamus, and frontal and temporal cortices.

**Figure 3:**
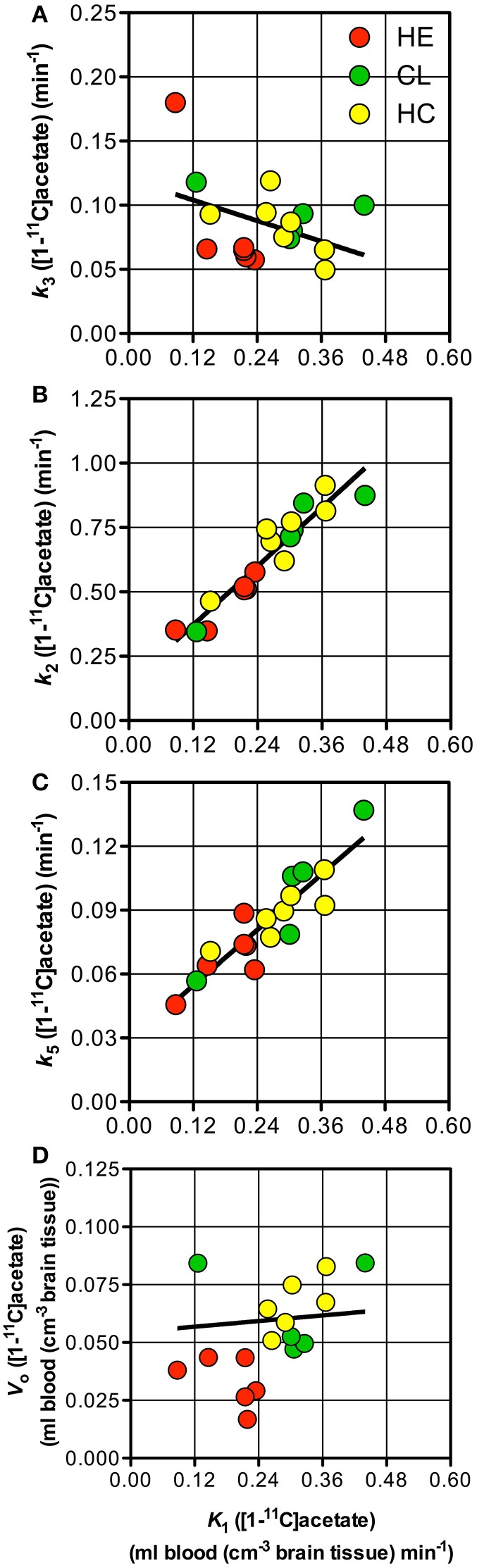
**Individual parameter estimates**. Patients with cirrhosis and hepatic encephalopathy (HE) (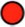), patients with cirrhosis without HE (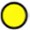), and healthy subjects (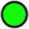). **(A)** Rate constant of whole brain astrocyte oxidative metabolism, *k*_3_vs. unidirectional clearance from blood-to-brain tissue of [^11^C]acetate, *K*_1_. **(B)** Rate constant for return of [^11^C]acetate from the astrocytes to blood, *k*_2_ vs. *K*_1_. **(C)** Passive diffusion of [^11^C]CO_2_ from the astrocytes into the blood, *k*_5_ vs. *K*_1_. **(D)** Apparent distribution volume in vascular space, *V*_0_ vs. *K*_1_. Solid line indicates a tendency.

Whole-brain individual estimates of *K*_1_ and *k*_3_ of [^11^C]acetate were not significantly correlated (Figure 3A). In contrast, whole-brain individual estimates of *K*_1_ were significantly correlated to *k*_2_ and *k*_5_ (both *P* < 0.0001), but not to *V*_0_ (*P* = 0.82) as shown in (Figures 3B–D).

The group of patients with cirrhosis and HE had significantly lower whole-brain and gray-matter metabolic clearances *K* of [^11^C]acetate than healthy subjects (Table 2). White matter had significantly lower values of *K* in all three groups of subjects compared to whole-brain and gray matter regions (Table 2), but the decline was less pronounced in the group of cirrhosis patients with HE.

## Discussion

**Figure 4:**
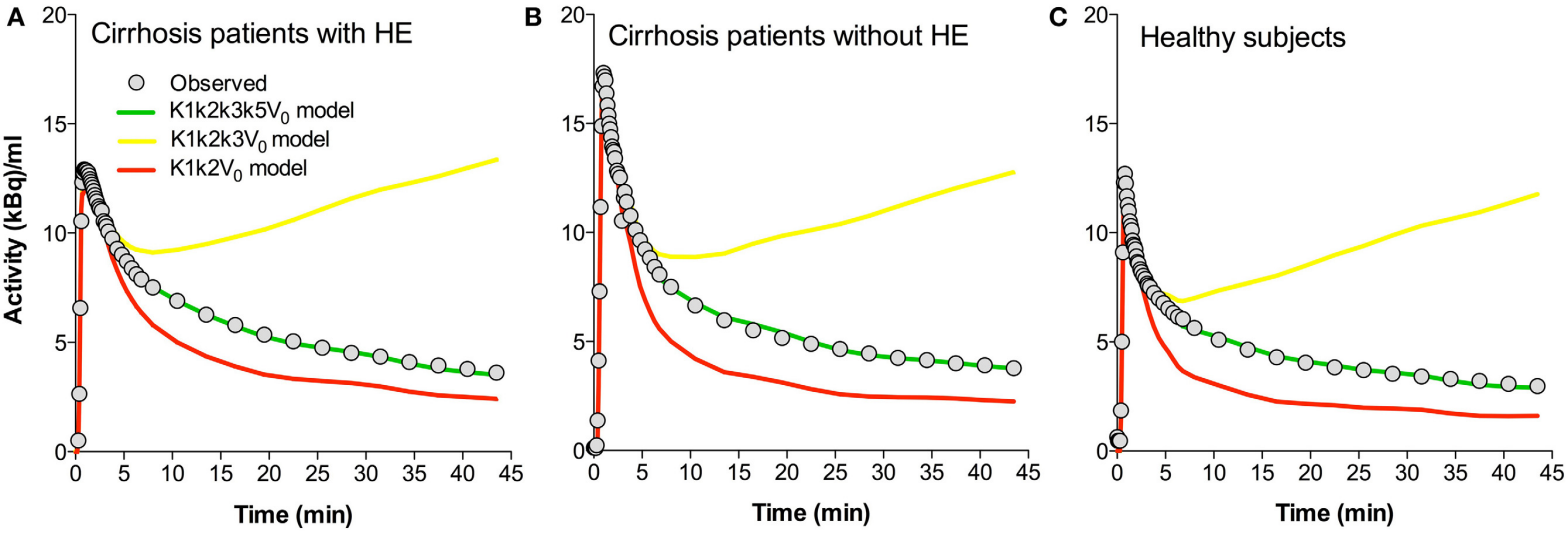
**Predicted time-activity curves for three whole-brain model configurations**. **(A)** Average data sets for patients with cirrhosis and hepatic encephalopathy (HE). **(B)** Patients with cirrhosis without HE. **(C)** Healthy subjects. Each panel shows the observed values, the fitted values using a *K*_1_*k*_2_*k*_3_*k*_5_V_0_ model (“full” model), and the predicted behaviors of the *K*_1_*k*_2_*k*_3_V_0_ and *K*_1_*k*_2_V_0_ models. *K*_1_, unidirectional clearance from blood-to-brain tissue of [^11^C]acetate. *k*_2_, rate constant for return of [^11^C]acetate from the astrocytes to blood. *k*_3_, rate constant of whole brain astrocyte oxidative metabolism. *k*_5_, passive diffusion of [^11^C]O_2_ from the astrocytes into the blood. *V*_0_, apparent distribution volume in vascular compartment.

In this study we fitted a novel model of [^11^C]acetate metabolism in brain tissue to brain [^11^C]acetate PET recordings. Figure 4 presents the time courses of the whole-brain [^11^C]-concentrations observed and those predicted by three different models for each of the three groups of subjects. The models include a simple flow model (*K*_1_, *k*_2_, *V*_0_), an irreversible metabolism model (*K*_1_, *k*_2_, *k*_3_, *V*_0_), and the current model with metabolite efflux (*K*_1_, *k*_2_, *k*_3_, *k*_5_, *V*_0_). The different time courses illustrate the discrimination among the predictions of the three formulations of the model, i.e., with the rate constants set to zero or not. We note that the present model fitted the data best.

**Figure 5:**
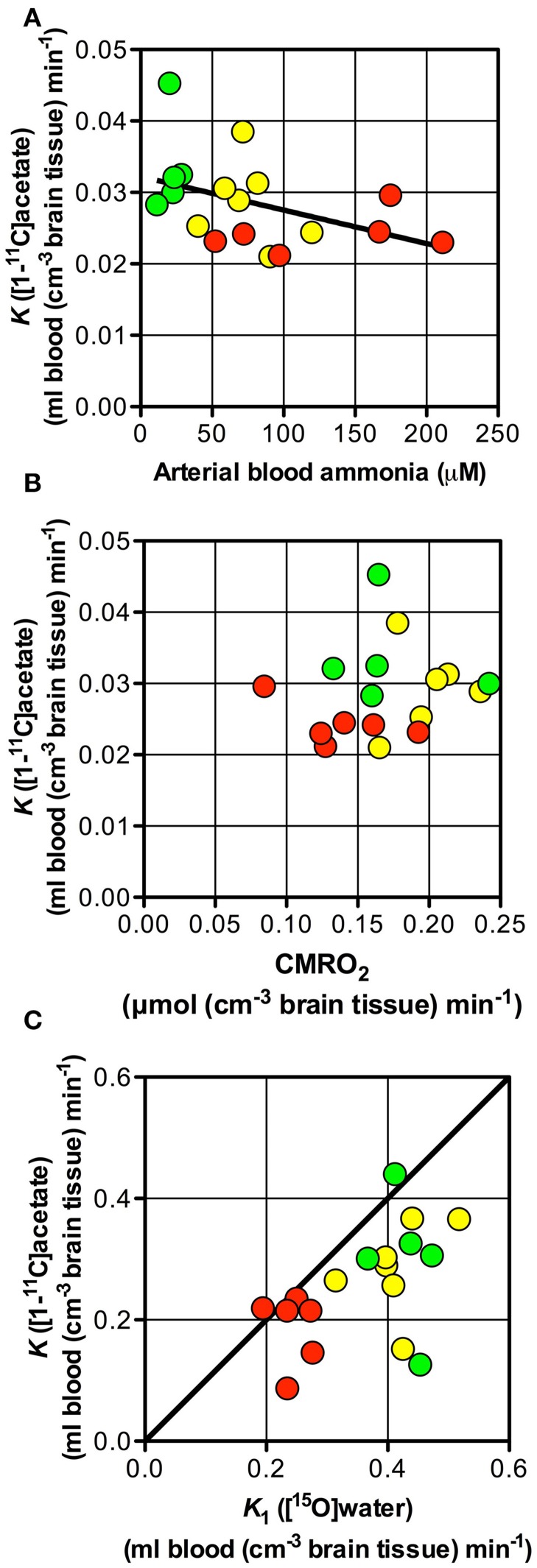
**Net metabolic clearance and unidirectional clearance from blood-to-brain tissue of [^11^C]acetate vs. arterial blood ammonia, unidirectional clearance of H_2_[^15^O] and cerebral metabolic rate of oxygen**. Patients with cirrhosis and hepatic encephalopathy (HE) (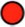), patients with cirrhosis without HE (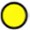), and healthy subjects (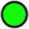). **(A)** Net metabolic clearance of [^11^C]acetate, *K* vs. arterial blood ammonia concentration (the latter from Iversen et al., 2009). **(B)** Net metabolic clearance of [^11^C]acetate, *K* vs. cerebral metabolic rate of oxygen, CMRO_2_ (the latter from Iversen et al., 2009). **(C)** Unidirectional clearance from blood-to-brain tissue of [^11^C]acetate, *K*_1_ vs. that *K*_1_ of H_2_[^15^O] (the latter from Iversen et al., 2009); the straight line shows line of identity. Solid line indicates a tendency.

The rate constant of oxidation of [^11^C]acetate in astrocytes (*k*_3_ of [^11^C]acetate) was not significantly different between the three groups of subjects (Table 2) and the net clearance of [^11^C]acetate (*K)* was not related to the arterial blood ammonia concentration or to the rate of oxygen consumption (Figures 5A,B). A possible explanation could be relatively lower aerobic glycolysis during HE, in which the consumption of glucose is replaced by acetate, with lower generation of lactate at normal brain tissue oxygen tensions (Gjedde et al., 2010). Lower generation of lactate in oligodendrocytes, lower blood flow, and up-regulation of the MCT1 transporters have been seen in other conditions, such as induced ischemia and epilepsy (Tseng et al., 2003; Rinholm et al., 2011).

The close correlation between *K*_1_of [^11^C]acetate and CBF (calculated as *K*_1_ of H^15^_2_O/0.85) (Iversen et al., 2009) as seen in Figure 5C, suggests that [^11^C]acetate uptake from blood is flow-determined by virtue of the great capacity of acetate transport by MCT1 across the blood-brain barrier and from the interstitial space into the astrocytes. The position of the observations just below the line of identity indicates an extraction fraction of [^11^C]acetate that is not much lower than that of [^15^O]water. This indicates that estimates of the *K*_1_ of [^11^C]acetate might be used as indices of CBF. Significant correlations of the estimates of *K*_1_ to *k*_2_ (*P* = 0.0001) and *k*_5_ (*P* = 0.0001), respectively (Figures 3B,C), can be explained by flow-dependence of *K*_1_, *k*_2_, and *k*_5_.

The higher *k*_3_/*k*_5_ ratio in patients with HE than in the two other group of subjects (Table 2) suggests that the oxidative metabolism of the astrocytes declines less than the rates of metabolite washout by the blood flow in patients with HE compared to subjects without HE.

Figure 2 shows all species of radioactivity within each of the three groups of subjects. The kinetic model deciphers the separate populations of labeled molecules, and their dynamic relationships, shown here to reveal similar fractions of the acetate pools being converted to substrates of the TCA cycles of the three groups. Of course, if significant differences of pharmacologically active pools of acetate existed, they would be reflected in different metabolic fluxes of acetate, but here we deal exclusively with tracer amounts of acetate, used solely to determine the enzyme activity of conversion to Acetyl-CoA in the three groups. There is practically no unlabeled acetate present. Of course, when ammonia concentrations differ, their metabolic fluxes would differ in proportion to the concentration differences.

The model used in this study is more complex than previously published models of Wyss et al. (2009), Lanz et al. (2012), emphasizing the observation that the previous models had only two parameters (*K*_1_ and *k*_2_) compared to the current model with five parameters (*K*_1_, *k*_2_, *k*_3_, *k*_5_, *and V*_0_). Wyss et al. (2009) interpret the clearance of activity (*k*_2_ in their model) as loss of [^11^C]CO_2_ generated by glial oxidation, and they found this not to be affected by increased CBF in their rat model. It is unclear how the (Wyss et al., 2009) model would account for the clearance of acetate as independent from the clearance of CO_2_. In the present model, *K*_1_, *k*_2_, and *k*_5_ all depend strongly on the rate of blood flow, *K*_1_ as a clearance, *k*_2_ and *k*_5_ as fractional clearances.

Astrocytic oxidative metabolism is generally held to be of the order of 20% only of the total brain oxidative metabolism (Hyder et al., 2010). In the present study. the blood flow reduction is likely to be the consequence rather than the cause of low oxygen metabolic rate is compromised, as previously reported (Gjedde et al., 2010).

The passive diffusion of monocarboxylic acids is known to be considerably lower than the facilitated diffusion across endothelial and other cell membranes (Cremer et al., 1979). Indeed, the actual uptake into neurons is much less than into astrocytes because of deficient facilitated rather than passive diffusion in the present study. In addition, the *k*_3_ magnitudes were shown not to differ, regardless of how large a proportion of the activity of the acetyl-CoA synthetase, estimated in the present study, actually reflected action in neurons.

In the HE condition, brain content of glutamine is likely to be elevated and could a potential confounder in the current study. The so-called “trojan horse” hypothesis claims that the enlarged glutamine pool is destructive to astrocytes, which would tend to be at variance with current evidence (Brusilow et al., 2010) and that the rate of oxidation of labeled acetate (*k*_3_) is unchanged in the present study.

As far as we know, the current study is the first to test and model a pathological state using [^11^C]acetate PET in humans, except for the one PET study with [^11^C]acetate of acute alcohol intoxication by Volkow et al. (2013), which did not report a detailed kinetic model. In previous studies, we demonstrated that ammonia has no inhibitory effect on the tricarboxylic acid cycle activity in cultured astrocytes (Johansen et al., 2007; Bak et al., 2009). In the same studies TCA cycle of cultured neurons (mainly glutamatergic) were not inhibited by ammonia as well. Thus, reduction of neuronal oxidative metabolism due to increased GABAergic tone (Hyder et al., 2006) could be an indirect mechanism by which increased ammonia reduces whole-brain oxidative metabolism of neurons in this condition (Gjedde et al., 1978, 2010; Leke et al., 2011; Schousboe et al., 2014).

In conclusion, the present study does not support the working hypothesis that the reduction in CMRO_2_ during HE in patients with cirrhosis reflects reduced oxidative metabolism by astrocytes. Hence, the reduced CMRO_2_ during HE is likely to be related to metabolic effects of the condition of HE in neurons.

## Financial support

Danish Council for Independent Research, Medical Sciences (09-065565) and the A. P. Møller Foundation for the Advancement of Medical Sciences (080255).

### Conflict of interest statement

The authors declare that the research was conducted in the absence of any commercial or financial relationships that could be construed as a potential conflict of interest.
